# Utilizing a Virtual Reality Matrix in Medical Education

**DOI:** 10.7759/cureus.66446

**Published:** 2024-08-08

**Authors:** Dipal Mistry, Sarah Geevarughese, Callaham A Brock, Jacob Nelson, Danielle Drew, Chris M Goddard, Ryan T West, Kyle Kleiman, Tom Lindsey

**Affiliations:** 1 Clinical, Biomedical, and Educational Research, Edward Via College of Osteopathic Medicine, Spartanburg, USA; 2 College of Medicine, Edward Via College of Osteopathic Medicine, Spartanburg, USA; 3 Surgery, Edward Via College of Osteopathic Medicine, Spartanburg, USA; 4 Family Medicine, Edward Via College of Osteopathic Medicine, Spartanburg, USA

**Keywords:** product selection matrix, simulation technology, product selection, matrix, virtual standardized patient, innovation in medical education, virtual reality in medical education, simulation in medical education, medical education

## Abstract

Virtual reality (VR) is an emerging technology that has demonstrated incredible value within medical education. However, medical institutions adopting VR as a learning tool need to ensure that the immersive technology product they pick possesses standard usability criteria. The current literature is limited in defining what specific criteria institutions should look for, or how to select between various VR products. Since there have been little to no algorithms available to the medical education community to aid in this process, a reproducible matrix has been developed to evaluate multiple VR platforms at once which can help identify the best option for medical education programs. The matrix is a 10-point scoring system that includes what the research team considered to be the 10 most important factors when selecting a VR product for medical education. The scores of any two or more VR products can be quantitatively compared. Therefore, the matrix is to be used as a methodological framework to help objectively select the highest-rated immersive technology platform. The research team involved in the development of the matrix consisted of an associate dean for simulation and technology, a director of simulation and technology, and eight medical students.

## Introduction

Virtual reality (VR) technology enables users to explore and interact with computer-generated three-dimensional environments, whether real or artificial, in real-time. This immersive experience allows users to acquire practical knowledge that can be applied in clinical practice. The use of VR in medical education is an emerging adjunct to traditional instructional methods. In recent years, medical education has made great strides in the incorporation of technological advancements into its highly structured and rigorous curriculum. The rise of VR has demonstrated remarkable potential for use in medical education and training [1]. Accordingly, VR is being increasingly integrated into medical education across all medical disciplines and at every level, from students to experienced practitioners. However, there is limited data on cohesive criteria used for the selection of a VR system to be used to meet the educational needs of a medical education program. Most conventional selection matrices used to evaluate products, such as the Analytical Hierarchy Process and the Technique for Order of Preference by Similarity to Ideal Solution, lack criteria that are unique to VR systems [2,3]. Consequently, there is no widely available standardized matrix used for comparison of VR product selection for medical education programs to best fit their specific needs [4].

A clear need was identified for a systemized list of distinct criteria that VR programs would need to meet to be successful in medical education. A reproducible 10-point matrix has been developed to evaluate multiple VR platforms to identify the best options for medical education programs. While a specific product is not reviewed, this report describes 10 points that delineate a comprehensive overview of a VR program that is both practical and applicable to medical education.

## Technical report

Methods

Introducing new technology into the medical school curriculum for educational purposes is not a light task. Whether it’s implementing a simple 2D web-based platform or a high-fidelity mannequin, both faculty and students need to agree that the technology is user-friendly. This is particularly true when wanting to introduce VR as a learning tool. The immersive technology is drastically different from standard graphical user interfaces, and thus its usability requires different criteria to be judged [3]. VR usability testing assesses users' ability to navigate, comprehend, and derive enjoyment from the VR experience, while also examining their handling of challenges such as motion sickness, disorientation, and fatigue. A feeling of immersion in the 3D virtual environment enhanced usability positively. Conversely, challenges with control, navigating within VR, and unfamiliarity had a detrimental effect on usability. If a usability problem is established, the technology is essentially a worthless investment for the institution as it likely actually impedes the learning process. Knowing this, the research team first searched the literature for previous ways VR technology used in a medical education setting was assessed/graded for its usability. To ensure a comprehensive literature review, we searched the PubMed, Google Scholar, EMBASE, and Cochrane Library databases using keywords related to using a matrix in medical education. To ensure a comprehensive literature review, the team broke into pairs with each group responsible for exploring one designated search engine. Keywords used during database searches included: Virtual Reality, Simulation, Medical Education, Standardized Patient, Product Selection, Evaluation, Algorithm, Matrix, and Criteria.

All papers reviewed were published in the English language without publication date cut-offs. Limited information was present from these database searches due to the paucity of literature on matrix development for VR products in medical education. Since the literature review lacked any articles specific to matrices for VR in medical education, the research team designed one.

Matrix design

**Figure 1:**
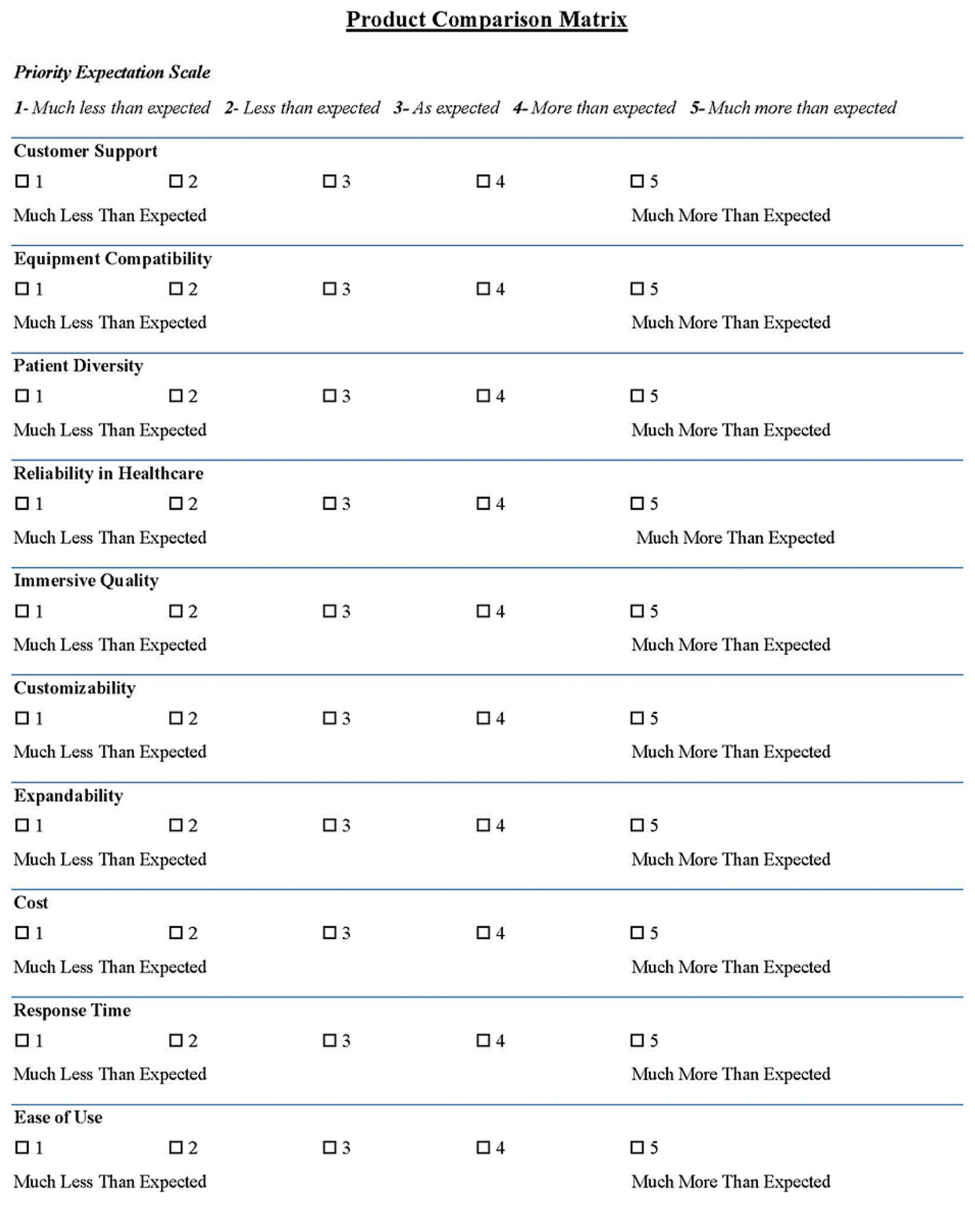
A 10-point matrix scale template serving as a platform to assist in the selection of virtual reality products for medical education. Image credit: Cal Brock, Danielle Drew, Sarah Geevorghese, Chris Goddard, Kyle Kleiman, Jacob Nelson, Dipal Mistry, and Ryan West

The research team involved in the development of the matrix consisted of an associate dean for simulation and technology, a director of simulation and technology, and eight medical students. The matrix is a 10-point scoring system that includes what the research team considered to be the 10 most important factors when selecting a VR product for medical education. These factors were determined by using the team’s prior experience with VR, speaking with representatives of various medical simulation platforms, and demonstrating a thorough analysis of currently published research in order to create a successful VR platform. The categories were then voted on among the team and a 10-point list was created. The following are the 10 factors that make up the matrix: (1) customer support, (2) equipment compatibility, (3) patient diversity, (4) reliability in healthcare, (5) immersive quality, (6) customizability, (7) expandability, (8) cost, (9) response time, and (10) ease of use. The numbering of this list has no ranking of importance. For example, (1) “customer support” is not weighted with more significance than (10) “ease of use.” Each of the 10 categories was then scored on a 1-5 point scale titled “Priority Expectation Scale.” The following represents the assigned point value: 1 - Much less than expected; 2 - Less than expected; 3 - As expected; 4 - More than expected; 5 - Much more than expected. Therefore, for any given company the minimum score possible was 10, while the maximum score possible was 50. Refer to Figure 1 for the matrix template. The scores of any two or more VR products can then be compared. This methodological framework serves as an objective guide to selecting the highest-scoring platform.

Matrix in action

First, it’s important to only look at platforms that focus on using immersive technology for medical education. For example, company X would be excluded if it profited, or marketed, from its software having an additional use case outside of medical education. This serves as a preliminary narrowing of programs that are then eligible to be compared against each other within the matrix. After selecting the eligible VR platforms (three or more are suggested) the matrix can then be applied.

Two or more raters within the team should independently evaluate the same platforms or assign specific matrix items to specialist raters within the team, in order to encompass diverse areas of expertise that may not be equally understood by every member of the team. Designate each member of the team to one or two of the eligible platforms. Each team member then initiates the evaluation by making contact with the company, scheduling demos, and doing general web-based reviews of their product. All findings from each company should then be presented to the whole team, being sure to highlight their software’s capabilities within the scope of the matrix’s categories. After each presentation, all members of the team use the 10-point matrix to score the company. After all platforms have been presented and scored, the data is then collected and compared. The VR company that has the highest score of a total of 50 points is deemed the best platform and should be pursued. The same matrix should be reviewed annually to incorporate upgrades and new software, ensuring the chosen product remains the best fit by evaluating essential criteria. If a superior alternative is identified through the matrix, then action to implement the product should be demonstrated. As new updates and new software are continuously occurring, this will guarantee that the medical school is investing in and delivering the most suitable product for their educational goals each academic year. This should not create a significant increase in cost as VR requires updates to maintain the accuracy, functionality, and compatibility of content and devices.

Matrix factors

Customer Support

Customer support is an integral component to consider when evaluating different pieces of new technology to adapt to an ecosystem [5]. Effective customer support includes appropriate response times based on the classification of the request (high, medium, or low priority). High-quality customer support is crucial during the implementation phase of adapting the new technology into the medical education ecosystem [6]. In addition, high-quality customer support is also important in the customizability aspect, whether a support team helps customize or you customize in-house. Therefore, customer support is a critical component to evaluate when deciding on a medical simulation technology to implement.

Equipment Compatibility

The ability to use software on many devices allows for many teams to easily adapt and implement this new technology. Implementing the technology to use current equipment (that already may be in the institution) or find the most appropriate equipment to meet the demands, costs, and requirements of the ecosystem where the technology is being implemented [4]. Therefore, equipment compatibility is a critical component to evaluate when deciding on a medical simulation technology to implement.

Patient Diversity

Considerations of patient diversity are essential to providing a well-rounded medical education for culturally competent medical students. A few elements of diversity, equity, and inclusion (DEI) that should be considered are the following: gender, race, ethnicity, sexual orientation, age, socioeconomic class, status of disability, etc. When providing medical education that considers these dimensions of diverse patient populations, there is a value that exceeds the academic setting [7]. These considerations refine the humanistic skills of medical students in preparation for the clinical setting.

Reliability in Healthcare

Reliability in healthcare is a valuable marker to assess a VR platform. As VR has been implemented in various institutions, it provides valuable feedback to evaluate the role such VR systems have had in improving medical education at that institution [5]. Quantitative metrics on the improvements students exhibit in classroom and clinical objectives offer insight into how a VR platform can be utilized and customized to meet the particular needs of a medical program.

Immersive Quality

The immersive quality was graded on three components: sense of presence, digital realism, and picture clarity. Sense of presence was influenced by sounds and haptics. Haptics are tactile responses when engaging with the virtual environment [8]. Digital realism can be enhanced by the interactivity of the virtual environment in which the user's actions within the virtual environment are immediately visible as well as conform to the laws of physics and the user’s perceptual expectation [3]. Picture clarity was measured by graphic qualities, such as high resolution, low latency, object texture, space, depth, contrast, and ability to manipulate the environment.

Customizability

Customizability is being able to modify the virtual environment to cater to your institution’s objectives [1]. This component allows customers to gain control of their investments by creating individualized and purposeful cases to deliver to their students. Questions to consider: Does the platform offer the ability to add curriculum-specific content? Or does the platform run off of internally produced cases?

Expandability

Expandability is the possibility of adding extra functionality to the original purchase, specifically related to the technical and physical properties of the program. For example, a program originally purchased for use in standardized patient encounters might have an extra function as a surgical training device. This would be considered expandable. Another example of an expandable program would be one that offers augmented reality with the ability to eventually add 3D capability. A more expandable platform would receive a higher score on this matrix.

Cost

Cost is an important parameter to consider when making a purchase [4]. This subsection examines the purchase price and compares the monetary cost of each potential purchase. A score of 3 would be the “expected” cost for an average product in that space (in our example, an average VR simulation program). A score of 1 would be an incredibly cheap program, while 5 would be among the most expensive. It is important to not conflate cost with value: the perceived value should not be considered in this section. It is strictly a comparison of costs.

Response Time

Response time can be thought of as the delay between the action of the user and the reaction of the VR platform. An optimal VR experience is created when there is minimal delay. A delay that is too high will cause the user to reject the simulation and the overall experience. It has also been shown that a high delay can lead to VR/simulator sickness which includes the symptoms of nausea, dizziness, and headache [6]. Due to the inherent nature of the brain to detect small amounts of delay, it is imperative that users choose a VR platform that minimizes delay.

Ease of Use

Ease of use can be equated to the intuitiveness of the components of the VR platform. Functionality within a given platform should not require extensive training or instruction [9]. Two outcomes will likely occur if large amounts of time and concentration have to be given to understanding the general functionality of a VR platform. One, the end user will likely not use the platform more than once. Two, the end user will likely not be able to utilize the full capabilities of the platform in an efficient manner. When comparing products regarding this quality it is important to measure how much effort is required to learn and then use the product [3].

## Discussion

With the recent history of COVID-19 and significant changes in the medium through which medical education is delivered including simulation technology, creating an easy-to-use matrix for selection of medical education technologies has never been more relevant [10]. The matrix delineated above offers both a comprehensive and adaptable platform in which to evaluate VR programs’ effectiveness within the context of medical education [10]. This approach to evaluation offers both a standard in which to quantitatively rank programs as well as flexibility regarding the broad scope of each matrix point. This was intentionally done to allow for the constant expansion and refinement of VR programs, thus alleviating the current deficit of such a tool in medical education [3]. The adaptability of this matrix allows medical education programs to apply this system not only in initiating a partnership with a VR platform but also to continue to assess its effectiveness and utility within their educational paradigm [11]. We suggest using this matrix combined with student feedback at least yearly to adequately assess the chosen VR program and make changes accordingly based on student response. In regard to the strengths of this matrix, it offers a clear and concise method to measure the effectiveness of a VR program in medical education. The matrix is easy to follow, and can therefore be viewed as an obtainable tool in practice. In regard to the weaknesses of this matrix, there is limited data at this time on its effectiveness in medical education as an objective measure. However, this leaves room for further study. More specifically, medical education programs may utilize this matrix to seek feedback from their students regarding the platform and may continuously assess for areas of improvement [12]. This fosters the advancement and innovation of medical education, quality improvement, and the ever-changing landscape of medicine.

## Conclusions

With rapid advancements in VR technology and the emergence of a wide variety of VR programs, it can be a daunting task for a medical education program to prioritize its specific needs when comparing multiple software programs. Through the development and implementation of a standardized, reproducible 10-point matrix for selecting VR software, we hope to assist medical education programs in their software selection. This matrix helps fill the gap in the current literature on criteria that could be considered when choosing between multiple VR programs for medical education programs. With the future landscape being favorable for increasing the incorporation of VR in medical education, this matrix will be critical in organizing the needs of any organization to select the best possible fit for their program.
